# Hypercalcemia caused by humoral effects and bone damage indicate poor outcomes in newly diagnosed multiple myeloma patients

**DOI:** 10.1002/cam4.3594

**Published:** 2020-11-04

**Authors:** Li Bao, Yutong Wang, Minqiu Lu, Bin Chu, Lei Shi, Shan Gao, Lijuan Fang, Qiuqing Xiang

**Affiliations:** ^1^ Department of Hematology Beijing Jishuitan Hospital Beijing China

**Keywords:** hypercalcemia, multiple myeloma, osteolysis, parathyroid hormone

## Abstract

**Background:**

Hypercalcemia of malignancy (HCM) is a serious metabolic complication, and the highest rates are in multiple myeloma (MM). The cause of hypercalcemia in newly diagnosed multiple myeloma (NDMM) remains unknown. We sought to evaluate the prognostic impact and mechanism of hypercalcemia in patients with symptomatic NDMM.

**Methods:**

We studied all consecutive MM patients who were initially diagnosed and followed up at Beijing Jishuitan Hospital between February 2013 and December 2019; 357 patients were included in the retrospective analysis.

**Results:**

A total of 16.8% of MM patients presented with hypercalcemia at the time of MM diagnosis. The presence of hypercalcemia was associated with higher serum levels of β2 microglobulin, creatinine, phosphorus, uric acid, procollagen I N‐terminal peptide, β‐carboxy‐terminal cross‐linking telopeptide of type I collagen and osteocalcin, lower serum levels of hemoglobin, parathyroid hormone (PTH), and advanced ISS and R‐ISS stages. Multivariate analysis showed that serum PTH, hemoglobin, creatinine, and uric acid levels were the main factors affecting hypercalcemia. The presence of hypercalcemia was associated with significantly inferior survival (40 months vs 57 months, *p* < 0.05) based on univariate analysis, and it remained an independent poor prognostic factor (HR: 1.854, 95% CI: 1.006‐3.415, adjusted *p* = 0.048) in a multivariate model that included age and R‐ISS stage.

**Conclusion:**

This study shows that hypercalcemia is associated with poor survival and is caused by manifold factors with humoral effects and local bone destruction.

## Graphical abstract

This study has shown that hypercalcemia is associated with poor survival and is caused by manifold factors with humoral effects and local bone destruction.

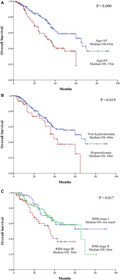

## 1 INTRODUCTION

Hypercalcemia of malignancy (HCM) is a serious metabolic complication, with the highest rates reported in multiple myeloma (MM).^1^, ^2^ HCM occurs as the result of humoral mechanisms, such as parathyroid hormone‐related protein (PTHrP)‐ or 1,25‐dihydroxy vitamin D (1,25(OH)2D)‐mediated pathways and direct bone metastasis.^3^, ^4^ Local osteolytic bone lesions are generally believed to be the main reason for hypercalcemia in patients with MM, and humoral hypercalcemia predominantly occurs in patients with squamous cell, renal cell, or ovarian cancer.^5^, ^6^, ^7^, ^8^, ^9^, ^10^, ^11^, ^12^, ^13^ Nevertheless, little is known about the proportion of hypercalcemia caused by HHM or local osteolysis in MM. Indeed, literature on the association between hypercalcemia and bone metabolism in patients with MM is lacking. In the older Durie and Salmon staging system, hypercalcemia was associated with a poorer prognosis,^14^ but it was not included in the international prognostic scoring system (ISS) or the revised ISS (R‐ISS) system.^15^, ^16^ Recently, Zagouri et al showed that hypercalcemia remains a poor prognostic factor in the era of novel agents.^17^

In this study, we aimed to identify the main factors influencing hypercalcemia by examining clinical and mineral bone metabolism biochemical markers; we also analyzed the effect of hypercalcemia on the survival of patients with newly diagnosed multiple myeloma (NDMM).

## 2 MATERIALS AND METHODS

### 2.1 Patients

The retrospective analysis included 357 consecutive unselected newly diagnosed patients with symptomatic MM treated within the Department of Hematology in Beijing Jishuitan Hospital between February 2013 and December 2019. The median follow‐up time was 24 months (5‐86 months), with follow‐up ending in April 2020. Hypercalcemia was defined as a serum calcium level >2.75 µmol/L and anemia as a hemoglobin level <10.0 g/dL. Renal failure was defined as creatine ≥177 µmol/L. Bone lesions were defined as one or more osteolytic lesions identified by skeletal radiography, computed tomography (CT), 18F‐fluorodeoxyglucose positron emission tomography with computed tomography (PET‐CT), or magnetic resonance imaging (MRI) according to the International Myeloma Working Group (IMWG) criteria.^18^ The ISS classification was used for the staging of all patients; 257 of the cases were examined using fluorescence in situ hybridization (FISH). The R‐ISS was applied for a subgroup of 325 patients. An analysis of overall survival (OS) was also performed. The database used is maintained at the Department of Hematology in Jishuitan Hospital; approval from the Scientific Committee/Institutional Review Board of Alexandra Hospital was obtained for the publication of these data, which are available upon request. In this retrospective study, laboratory data (biochemical, hematological, and electrophoretic results) obtained at the time of diagnosis were extracted from patients’ medical records; the data were analyzed after patient anonymization.

### 2.2 Treatments

Patients up to 65 years of age and in good clinical condition received four cycles of bortezomib‐ or thalidomide‐based induction regimens, such as VAD, VTD, VCD, or TAD (VAD: bortezomib 1.3 mg/m^2^, administered intravenously on days 1, 8, 15, and 22; dexamethasone 40 mg, administered intravenously on days 1 through 4, 8, 15, and 22; pegylated liposomal doxorubicin 25 mg/m^2^, administered intravenously on day 1. VTD: bortezomib 1.3 mg/m^2^, administered intravenously on days 1, 8, 15, and 22; dexamethasone 40 mg, administered intravenously on days 1 through 4, 8, 15, and 22; thalidomide 100 mg po qn; VCD: bortezomib 1.3 mg/m^2^, intravenously on days 1, 8, 15, and 22; CTX 300 mg/m^2^, intravenously on days 1, 8, and 15; dexamethasone 40 mg, on days 1‐4, 8, 15, and 22. TAD: thalidomide 100 mg po qn; pegylated liposomal doxorubicin 25 mg/m^2^ on day 1; dexamethasone 40 mg on days 1‐4, 8, 15, and 22). Patients who achieved PR underwent stem cell mobilization with cyclophosphamide and granulocyte colony‐stimulating factor, followed by high‐dose melphalan and ASCT. Consolidation therapy typically consists of two to four cycles of a repeated induction regimen, which aims to improve the degree of response after transplant. Lenalidomide maintenance was considered to be post‐ASCT for all patients.

Among transplant‐ineligible patients, which are usually older and might be considered unsuitable owing to comorbidities, disability, or disease burden, they continuously received VD or a reduced‐intensity regimen of VAD or TAD and then lenalidomide maintenance for those with above VGPR.

Treatment options for refractory and relapsed cases include retreating with a previously used agent, switching to another agent in the same drug class, or switching to an agent in a different drug class. Additionally, BD‐PACE, RD‐PACE, BRD, MPV, and BTD were selected for salvage therapy of RRMM.

For patients with hypercalcemia, the conventional therapy included venoclysis of 5% glucose solution to hydration, sodium bicarbonate to alkalinize the urine and the use of a diuretic. Patients without renal dysfunction also received bisphosphonate treatment. The most effective treatment for MM is chemotherapy.

### 2.3 Assays

Blood samples for MM diagnosis in the study were collected within 72 hours. Drawn in the early morning on the hospital ward, all blood samples were analyzed at the Jishuitan Hospital Laboratory according to the manufacturer's protocol. Plasma P1NP, βCTX, Osteocalcin, total vitamin D, and PTH levels were measured using electrochemiluminescence immunoassays (Roche Diagnostics GmbH, Sandhofer Strasse 116). The inter‐/intra‐assay coefficients of variation in plasma βCTX was between 0.010 ng/ml and 6.00 ng/ml. The assay range of plasma P1NP was 5 ng/ml to 1200 ng/ml, that of plasma osteocalcin was 0.500 ng/ml to 300 ng/ml, that of plasma vitamin D total was 3.00 ng/ml to 70.0 ng/ml, and that of plasma PTH was 1.20 ng/ml to 5000 ng/ml.

### 2.4 Statistical analysis

Differences between mean values were evaluated by both Student's t and Mann–Whitney tests, and *p* values <0.05 were considered significant. Comparisons of categorical variables among different groups were carried out using the chi‐square test or Fisher's exact test when appropriate. Pearson correlation analysis was performed to assess linear correlation between two parameters. OS was measured from the date of treatment initiation until the date of death or the date of the last follow‐up. Time to event curves were plotted with the Kaplan–Meier method, and comparisons among groups were conducted using the log‐rank test. For multivariate analysis, factors associated with time to event were introduced into a Cox proportional hazards model. SPSS 18.0 software (SPSS, Inc., Chicago, IL, USA) was used for the statistical analysis. A *p*‐value of <0.05 was considered statistically significant.

## 3 RESULTS

### 3.1 Baseline characteristics of patients

**Figure 1:**
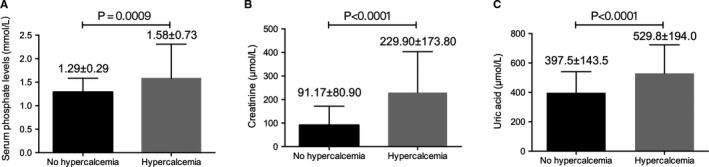
Increased serum phosphonate, creatinine, and uric acid levels correlate with hypercalcemia in MM patients (*p* < 0.001)

The baseline characteristics of the patients with NDMM are described in Table 1. Using the IMWG definition, 16.8% (60/357) of the patients with symptomatic MM had hypercalcemia (i.e., serum calcium ≥2.75 µmol/L) at the time of diagnosis. The presence of hypercalcemia was strongly associated with features of advanced disease, including β_2_ microglobulin, hemoglobin, creatinine, uric acid, phosphorus, ISS stage, and R‐ISS stage (*p* < 0.01 for all comparisons) (Tables 2 and 3 and Figure 1). Among the 357 patients, 52 (14.6%) had renal insufficiency, among whom 31 (59.6%) had hypercalcemia. In our study, 91.2% of patients had lytic bone lesions identified by imaging, such as MRI, CT, and PET‐CT. In the subgroup of patients with available cytogenetic data (N = 257), there were no differences between those with hypercalcemia and without hypercalcemia (Table 3).

**Table 1:** Patient characteristics

Characteristics	N.
No. of patients	357
Median age, range	60 (27‐89)
Male (%)	199 (55.7)
M protein (%)	
IgG	153 (42.8)
IgA	86 (24.1)
IgD	14 (3.9)
Light chain	94 (26.3)
No secreted	10 (2.9)
ISS (%)	
I	117 (32.8)
II	109 (30.5)
III	(131 36.7)
R‐ISS (%)	
I	71 (21.8)
II	193 (59.4)
III	61 (18.8)
Failed in Staging*	32 (8.9%)
Frequency of CRAB features (%)	
Hypercalcemia	60 (16.8)
Renal dysfunction	52 (14.6)
Anemia	219 (61.4)
Bone disease	325 (91.2)
Median follow‐up, months (range)	24 (1‐82)

Abbreviations: ISS, International staging system; RISS, revised international staging system; CRAB, features of MM patients, including hypercalcemia, renal dysfunction, anemia, and bone disease.

* 32 patients failed in FISH detection and unable to stage in RISS.

**Table 2:** Evaluation of biochemical indicators in the subgroup of consecutive patients with available data (n = 357)

	Patients with hypercalcemia (N = 60)	Patients without hypercalcemia (N = 297)	*p*‐value
Sex (male/female)	34/26	165/132	0.888
Age (median/range)	58 (41‐83)	61 (27‐89)	0.748
Hemoglobin, g/L	83 (48‐158)	109 (31‐174)	<0.001
β_2_ microglobulin, mg/L	7.175 (1.73‐54.38)	3.92 (1.13‐52.01)	0.002
Creatinine, umol/L	186 (54‐812)	71 (28‐1104)	<0.001
Phosphorus, mmol/L	1.38 (0.74‐5.59)	1.28 (0.56‐2.3)	<0.001
Uric acid, umol/L	522.5 (7‐1076)	378 (10‐803)	0.006
LDH, IU/L	166 (88‐488)	171.5 (38‐940)	0.244
ALP, IU/L	76 (30‐216)	70 (26‐640)	0.871
tP1NP, ng/ml	86.5 (2.45‐362)	63.47 (13.38‐546.5)	0.277
β‐CTX, ng/ml	2.22 (0.2‐5.29)	0.73 (0.07‐3.57)	0.001
OC, ng/ml	29.45 (7.25‐161.2)	18.56 (3.7‐190.7)	<0.001
25‐(OH)_2_D_3_, ng/ml	12.6 (3‐69.87)	15.33 (2.0‐47.26)	0.002
PTH, pg/ml	11.9 (3.8‐99)	28.2 (4.6‐20.3)	0.024

Abbreviations: ALP, Alkaline phosphatase; LDH, lactate dehydrogenase; OC, N‐terminal osteocalcin; PTH, parathyroid hormone; tPINP, total type 1 procollagen N‐terminal elongation peptide; β‐CTX, C‐terminal cross‐linking telopeptide β of type 1 collagen.

**Table 3:** Evaluation of cytogenetic factors and stages in the subgroup of consecutive patients with available data

	Patients with hypercalcemia, N = 60	Patients without hypercalcemia, N = 297	*p*‐value
del13q (by FISH)	18 (30.2)	95 (32.0)	0.860
amp1q21	14 (23.2)	62 (20.9)	0.838
del17p	12 (20.9)	54 (18.6)	0.831
t(4;14)	8 (13.9)	26 (8.8)	0.394
t(14;16)	4 (6.9)	18 (6.0)	1.0
t(11;14)	10 (16.3)	54 (18.1)	0.832
High‐risk FISH	25 (41.8)	107 (36.3)	0.605
ISS stage			0.000
I	4 (6.7)	98 (38.0)	
II	8 (13.3)	101 (34.0)	
III	48 (80)	83 (28)	
R‐ISS stage			0.000
I	1 (2.0)	66 (22.2)	
II	33 (55.1)	157 (52.8)	
III	26 (42.9)	42 (14.1)	
Failed in Staging *	0	32 (10.9)	

Data were shown in number (percentage).

Abbreviations: ISS, International staging system; RISS, revised international staging system.

* 32 patients failed in FISH detection and unable to stage in RISS.

### 3.2 Relationship between hypercalcemia and clinical and bone mineral metabolism markers

**Figure 2:**
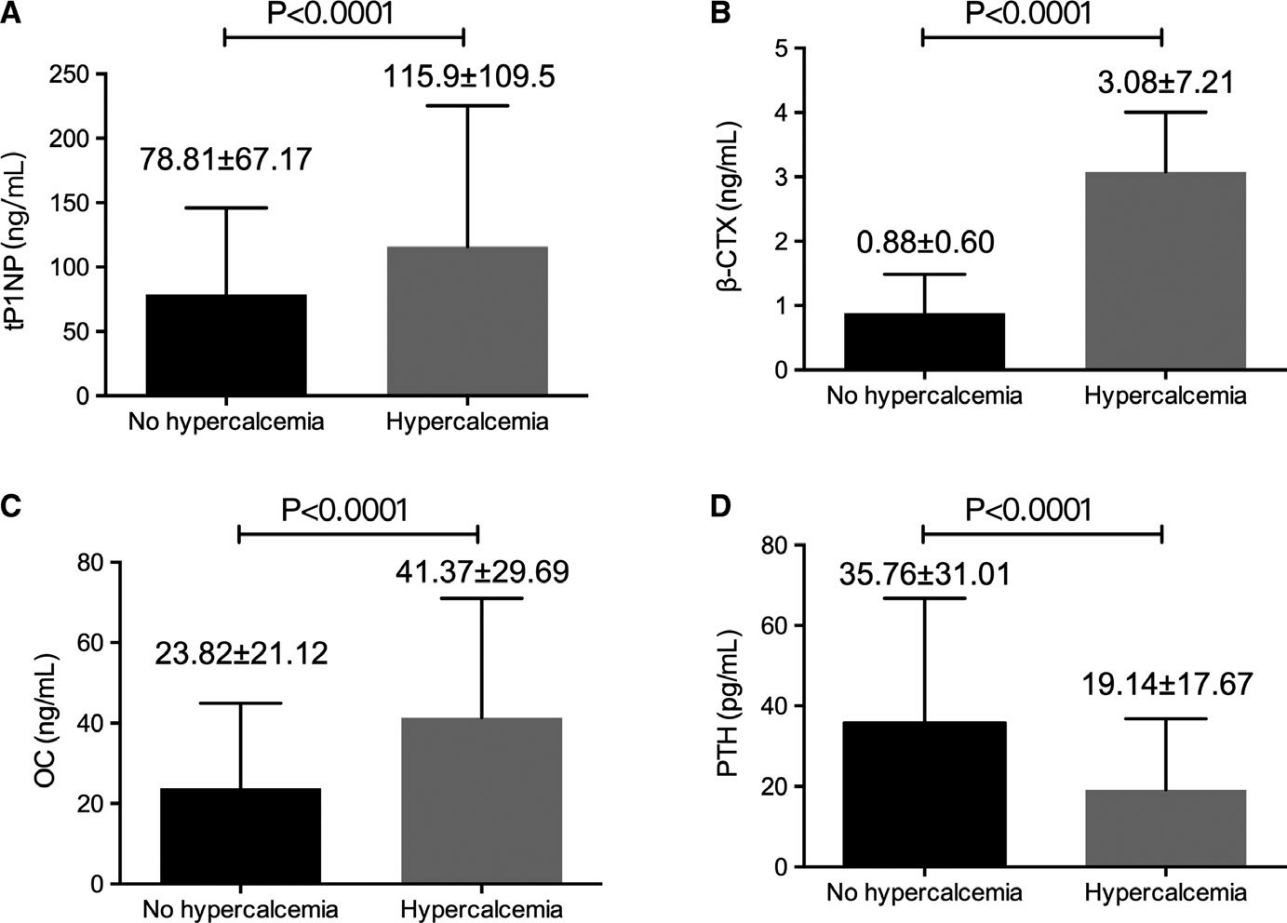
Increased serum tPINP, β‐CTX, and OC levels and decreased serum PTH level correlate with hypercalcemia in MM patients (*p* < 0.001)

Serum levels of β‐CTX, tPINP, and osteocalcin (OC) were significantly elevated but PTH levels significantly decreased in patients with hypercalcemia (Table 2, Figure 2). Serum alkaline phosphatase (ALP) and lactate dehydrogenase (LDH) levels were not associated with calcium abnormalities (Table 2). Furthermore, Patients presented of lytic bone lesion had a higher level of tP1NP than patients without osteolytic disease (68.25 ng/ml vs 50.04 ng/ml, *p* = 0.035, data not shown).

When multivariate analysis was performed using serum levels of hemoglobin, phosphorus, β‐CTX, tPINP, OC, 25‐(OH)_2_D_3_, PTH, creatinine, and uric acid, laboratory parameters (PTH, creatinine, uric acid, and hemoglobin) were found to be the most important factors of hypercalcemia (Table 4).

**Table 4:** Logistic regression of multivariate analysis for hypercalcemia

	Exp (B)	95% CI for EXP (B)		Sig
		Lower	Upper	
PTH	0.967	0.943	0.992	0.006
CRE	1.006	1.003	1.010	0.001
Uric acid	1.005	1.002	1.008	0.001
Hemoglobin	0.978	0.962	0.993	0.005

Abbreviations: CRE, creatinine; PTH, parathyroid hormone.

Among the 60 patients with hypercalcemia, mild hypercalcemia (serum calcium 11‐12 mg/dl, 2.75–3.0 mmol/L) accounted for 35%, moderate hypercalcemia (serum calcium 12–14 mg/dl, 3–3.5 mmol/L) for 46.7%, and severe hypercalcemia (serum calcium>14 mg/dl, >3.5 mmol/L) for 18.3%. The degree of hypercalcemia was closely associated with renal failure, and the proportion of mild, moderate, and severe hypercalcemia patients with renal failure was 23.8%, 60.7%, and 81.8%, *p* = 0.001, respectively (Table 5). Forty percent of patients with hypercalcemia had normal or mildly elevated PTH levels.

**Table 5:** Relationship between the degree of hypercalcemia and renal failure in 60 cases

Classification of hypercalcemia	n	Renal failure, n (%)	*p*
Mild (2.75‐3 mmol/L)	21	5 (23.8)	0.001
Moderate (3‐3.5 mmol/L)	28	17 (60.7)	
Severe (>3.5 mmol/L)	11	9 (81.8)	

### 3.3 Impact of hypercalcemia on prognosis

**Figure 3:**
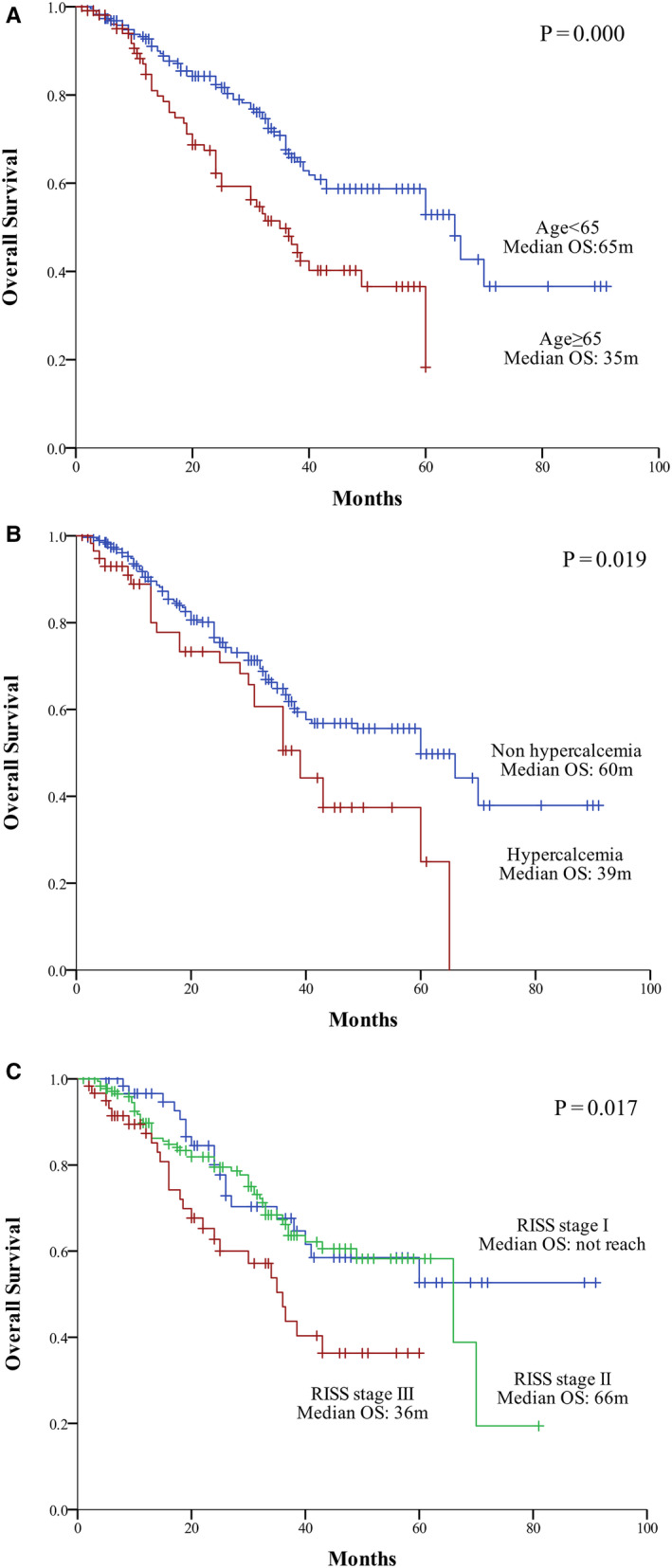
Survival comparison between multiple factors. (A) With age greater than or equal to 65 years or less than 65 years, the median OS was 65 months vs 35 months, respectively (*p* = 0.000). (B) With hypercalcemia and no hypercalcemia, the median OS was 60 months vs 39 months, respectively (*p* = 0.019). (C) With R‐ISS stage I, II, and III, the median OS was not reached at 66 months or 36 months (*p* = 0.017)

According to univariate analysis, the presence of hypercalcemia was associated with significantly inferior survival (36.3 months vs 56.8 months, *p* = 0.019). We examined the prognostic impact of hypercalcemia in a multivariate model that also included established prognostic factors (ISS stage, R‐ISS stage, age, high‐risk FISH findings), and hypercalcemia remained an independent poor prognostic factor (HR: 1.854, 95% CI: 1.006‐3.415, adjusted *p* = 0.048) (Table 6, Figure 3).

**Table 6:** Multivariate analysis for myeloma OS

Variable	Hazard ratio (HR)	95% CI for HR	*p*‐value
Age >65	2.628	1.559‐4.430	0.000
Hypercalcemia	1.854	1.006‐3.415	0.048
R‐ISS	1.952	1.254‐3.038	0.003

Early death occurred in 18.33% and 8.41% of patients with and without hypercalcemia, respectively (*p* = 0.048).

## 4 DISCUSSION

The bone remodeling process, intestinal absorption, and renal tubule resorption are the three main metabolic pathways that contribute to maintaining the serum calcium concentration in a narrow physiological range. Hyper‐ or hypocalcemia occurs due to imbalances in these regulatory pathways. HCM is the most common cause of hypercalcemia, which is a potentially life‐threatening but relatively common clinical problem.^19^, ^20^ Serum calcium is tightly regulated by interactions between PTH and (1,25(OH)_2_D), which modulate calcium movement at the level of the bone, kidneys, and intestines.^21^ Local osteolytic hypercalcemia and PTHrP‐ and/or 1,25(OH)_2_D‐induced disorder are the two main reasons for HCM. In patients with localized, as well as generalized, myeloma bone disease, bone destruction, together with a lack of bone formation, results in excess bone resorption and causes excessive release of calcium, leading to hypercalcemia.^22^ Osteoclast‐activating factors stimulate bone resorption through nuclear factor kappa B ligand (RANKL), macrophage inflammatory protein 1α, interleukin 6, and tumor necrosis factor α signaling, causing recruitment of additional osteoclasts and increased calcium release into the circulation.^23^, ^24^

Unfortunately, to date, there are no reports on the association between hypercalcemia and the amount or degree of bone damage in patients with MM. In our study, patients with bone destruction accounted for 91.2% of all patients; the vast majority of patients had more than 3‐5 lesions, though only 16.8% of patients had hypercalcemia. Thus, hypercalcemia mainly caused by localized bone disease could not well explain our clinical results. The presence of hypercalcemia was associated with higher serum levels of β‐CTX, which reflects bone resorption, or tPINP and OC, which reflect bone formation. Nonetheless, β‐CTX and OC were not significant factors in multivariate analysis. This result indicates that although local bone lesions are related to hypercalcemia, they are not the most important factors for hypercalcemia. We think that humoral hypercalcemia is still the main cause of hypercalcemia in MM. On the other hand, both 25‐(OH)_2_D_3_ and PTH levels were significantly decreased in patients with hypercalcemia compared with patients without hypercalcemia. Among the 357 patients with NDMM, 14.6% had renal failure, and 59.6% with renal failure had hypercalcemia. Serum levels of phosphorus, creatinine, and uric acid were significantly increased in patients with hypercalcemia compared with patients without hypercalcemia. PTH, hemoglobin, creatinine, and uric acid were the factors most closely related to hypercalcemia in multivariate analysis. Hypercalcemia may develop in patients with osteoporosis and treated with recombinant PTH (1‐84), and serum calcium correlates with urinary calcium excretion, serum ALP, and β‐CTX in these patients.^25^

In particular, kidney impairment accounted for 81.8% of patients with severe hypercalcemia (serum calcium >3.5 mmol/L), further indicating the important role of the kidneys in hypercalcemia. Due to the excessive deposition of immunoglobulin in patients with MM, renal tubular function is often impaired, which leads to increased calcium reabsorption by renal tubules. Subsequently, the ability of the kidney to effectively remove excessive calcium from the circulation is hampered, resulting in elevated serum calcium, which may lead to severe hypercalcemia and renal failure. In addition, hemoglobin had a faint effect on hypercalcemia, as shown in Table 3. We hypothesize that anemia is indirectly related to hypercalcemia, as hemoglobin correlated negatively with serum creatinine (*p* < 0.001, data not shown).

Overall, we think that although bone destruction is a characteristic change in MM, serum calcium is still regulated by internal secretion. Although the reasons for hypercalcemia are manifold, among them, humoral hypercalcemia may be highly important. Unfortunately, we only retrospectively analyzed bone turnover metabolism and biochemical markers and did not detect PTHrP. Despite the lack of PTHrP analysis, previous studies have confirmed that PTHrP produced by plasma cells in MM regulates their survival and pro‐osteoclast activity, promoting bone disease progression.^26^, ^27^, ^28^, ^29^, ^30^

The presence of hypercalcemia was strongly associated with features of advanced disease, such as β_2_ microglobulin, ISS stage, and R‐ISS stage. Our results are consistent with those of many previous studies showing that hypercalcemia is a factor for a poor prognosis.^31^, ^32^ However, the presence of hypercalcemia is not included in the ISS stage or the R‐ISS stage, though it was an inferior prognostic factor in the original dataset.^16^ In our study, we found that hypercalcemia, age greater than 65 years old, and R‐ISS stage were three independent prognostic factors affecting the survival of MM patients.

In our patient population, hypercalcemia was associated with a significantly higher early mortality rate than no hypercalcemia, with rates of 18.33% vs 8.41%. This result is consistent with that reported by Zagouri et al.^17^ Indeed, early mortality is a major barrier to the treatment of MM.^33^ Therefore, patients with a high risk of early mortality should be identified early and managed appropriately.

In our study, which was based on a single‐center database of consecutive, unselected patients, thalidomide‐containing or bortezomib‐based antimyeloma therapy was used in patients presenting with hypercalcemia. The symptoms and serum calcium level of all patients with hypercalcemia improved quickly within the first week after bisphosphonate application and chemotherapy. However, the use of these drugs did not alter the poor prognosis of patients with hypercalcemia.

## 5 CONCLUSIONS

Our study based on patients with symptomatic NDMM at a single center showed that hypercalcemia is associated with poor survival and is caused by manifold factors with humoral effects and local bone destruction.

## CONFLICTS OF INTEREST

The authors have no conflicts of interest to disclose.

## AUTHOR CONTRIBUTIONS

L. B designed the study; Y.‐T.W. collected the data; L. B analyzed the data and wrote the manuscript. All authors contributed to the interpretation of the data, prepared the manuscript, and approved the final version.

## Data Availability

The analysis data and models we used during the study are available from the corresponding author by request.

## References

[1] Dellay B , Groth M . Emergency management of malignancy‐associated hypercalcemia. Adv Emerg Nurs J. 2016;38(1):15‐25. quiz E1. 26817427 10.1097/TME.0000000000000093

[2] Mhaskar R , Kumar A , Miladinovic B , Djulbegovic B . Bisphosphonates in multiple myeloma: an updated network meta‐analysis. Cochrane Database Syst Rev. 2017;12:CD003188. 29253322 10.1002/14651858.CD003188.pub4 PMC6486151

[3] Stewart AF , Horst R , Deftos LJ , Cadman EC , Lang R , Broadus AE . Biochemical evaluation of patients with cancer‐associated hypercalcemia: evidence for humoral and nonhumoral groups. N Engl J Med. 1980;303(24):1377‐1383. 6253785 10.1056/NEJM198012113032401

[4] Burtis WJ , Brady TG , Orloff JJ , et al. Immunochemical characterization of circulating parathyroid hormone‐related protein in patients with humoral hypercalcemia of cancer. N Engl J Med. 1990;322(16):1106‐1112. 2320080 10.1056/NEJM199004193221603

[5] Burt ME , Brennan MF . Incidence of hypercalcemia and malignant neoplasm. Arch Surg. 1980;115(6):704‐707. 7387355 10.1001/archsurg.1980.01380060012004

[6] Vassilopoulou‐Sellin R , Newman BM , Taylor SH , Guinee VF . Incidence of hypercalcemia in patients with malignancy referred to a comprehensive cancer center. Cancer. 1993;71(4):1309‐1312. 8382106 10.1002/1097-0142(19930215)71:4<1309::aid-cncr2820710423>3.0.co;2-m

[7] Kyle RA , Gertz MA , Witzig TE , et al. Review of 1027 patients with newly diagnosed multiple myeloma. Mayo Clin Proc. 2003;78(1):21‐33. 12528874 10.4065/78.1.21

[8] Christoulas D , Terpos E , Dimopoulos MA . Pathogenesis and management of myeloma bone disease. Expert Rev Hematol. 2009;2(4):385‐398. 21082944 10.1586/ehm.09.36

[9] Roodman GD , Dougall WC . RANK ligand as a therapeutic target for bone metastases and multiple myeloma. Cancer Treat Rev. 2008;34(1):92‐101. 17964729 10.1016/j.ctrv.2007.09.002

[10] Stewart AF . Clinical practice. Hypercalcemia associated with cancer. N Engl J Med. 2005;352(4):373‐379. 15673803 10.1056/NEJMcp042806

[11] Sternlicht H , Glezerman IG . Hypercalcemia of malignancy and new treatment options. Ther Clin Risk Manag. 2015;11:1779‐1788. 26675713 10.2147/TCRM.S83681 PMC4675637

[12] Acikgoz Y , Sendur MA , Aksoy S , Ozdemir NY , Zengin N . Metastatic parenchymal renal squamous cell carcinoma with hypercalcemia. Med Oncol. 2014;31(9):169. 25134916 10.1007/s12032-014-0169-3

[13] Ngo N , Edriss H , Figueroa JA , Nugent K . Squamous cell carcinoma of the sigmoid colon presenting with severe hypercalcemia. Clin Colorectal Cancer. 2014;13(4):251‐254. 25444465 10.1016/j.clcc.2014.06.006

[14] Durie BG , Salmon SE . A clinical staging system for multiple myeloma. Correlation of measured myeloma cell mass with presenting clinical features, response to treatment, and survival. Cancer. 1975;36(3):842‐854. 1182674 10.1002/1097-0142(197509)36:3<842::aid-cncr2820360303>3.0.co;2-u

[15] Greipp PR , San Miguel J , Durie BG , et al. International staging system for multiple myeloma. J Clin Oncol. 2005;23(15):3412‐3420. 15809451 10.1200/JCO.2005.04.242

[16] Palumbo A , Avet‐Loiseau H , Oliva S , et al. Revised International Staging System for Multiple Myeloma: A Report From International Myeloma Working Group. J Clin Oncol. 2015;33(26):2863‐2869. 26240224 10.1200/JCO.2015.61.2267 PMC4846284

[17] Zagouri F , Kastritis E , Zomas A , et al. Hypercalcemia remains an adverse prognostic factor for newly diagnosed multiple myeloma patients in the era of novel antimyeloma therapies. Eur J Haematol. 2017;99(5):409‐414. 28675766 10.1111/ejh.12923

[18] Rajkumar SV , Dimopoulos MA , Palumbo A , et al. International Myeloma Working Group updated criteria for the diagnosis of multiple myeloma. Lancet Oncol. 2014;15(12):e538‐e548. 25439696 10.1016/S1470-2045(14)70442-5

[19] Lindner G , Felber R , Schwarz C , et al. Hypercalcemia in the ED: prevalence, etiology, and outcome. Am J Emerg Med. 2013;31(4):657‐660. 23246111 10.1016/j.ajem.2012.11.010

[20] Zagzag J , Hu MI , Fisher SB , Perrier ND . Hypercalcemia and cancer: differential diagnosis and treatment. CA Cancer J Clin. 2018;68(5):377‐386. 30240520 10.3322/caac.21489

[21] Horwitz MJ , Hodak SP , Stewart AF . Non‐parathyroid hypercalcemia In Rosen CJ, ed. Primer on the Metabolic Bone Diseases and Disorders of Mineral Metabolism. Scarborough, ME: Maine Medical Center Research Institute; 2008:307‐312.

[22] Walker RE , Lawson MA , Buckle CH , Snowden JA , Chantry AD . Myeloma bone disease: pathogenesis, current treatments and future targets. Br Med Bull. 2014;111(1):117‐138. 25190762 10.1093/bmb/ldu016

[23] Roodman GD . Mechanisms of bone metastasis. N Engl J Med. 2004;350(16):1655‐1664. 15084698 10.1056/NEJMra030831

[24] Choi SJ , Cruz JC , Craig F , et al. Macrophage inflammatory protein 1‐alpha is a potential osteoclast stimulatory factor in multiple myeloma. Blood. 2000;96(2):671‐675. 10887133

[25] Luna‐Cabrera F , Justicia‐Rull EA , Caricol‐Perez MP , et al. Incidence of hypercalcemia, hypercalciuria and related factors in patients treated with recombinant human parathyroid hormone (1–84). Minerva Med. 2012;103(2):103‐110. 22513515

[26] Cafforio P , Savonarola A , Stucci S , et al. PTHrP produced by myeloma plasma cells regulates their survival and pro‐osteoclast activity for bone disease progression. J Bone Miner Res. 2014;29(1):55‐66. 23787729 10.1002/jbmr.2022

[27] Tsujimura H , Nagamura F , Iseki T , Kanazawa S , Saisho H . Significance of parathyroid hormone‐related protein as a factor stimulating bone resorption and causing hypercalcemia in myeloma. Am J Hematol. 1998;59(2):168‐170. 9766803 10.1002/(sici)1096-8652(199810)59:2<168::aid-ajh11>3.0.co;2-5

[28] Ohmori M , Nagai M , Fujita M , et al. A novel mature B‐cell line (DOBIL‐6) producing both parathyroid hormone‐related protein and interleukin‐6 from a myeloma patient presenting with hypercalcaemia. Br J Haematol. 1998;101(4):688‐693. 9674742 10.1046/j.1365-2141.1998.00758.x

[29] Kitazawa R , Kitazawa S , Kajimoto K , et al. Expression of parathyroid hormone‐related protein (PTHrP) in multiple myeloma. Pathol Int. 2002;52(1):63‐68. 11940209 10.1046/j.1440-1827.2002.01314.x

[30] Otsuki T , Yamada O , Kurebayashi J , et al. Expression and in vitro modification of parathyroid hormone‐related protein (PTHrP) and PTH/PTHrP‐receptor in human myeloma cells. Leuk Lymphoma. 2001;41(3–4):397‐409. 11378553 10.3109/10428190109057995

[31] Yusuf AA , Natwick T , Werther W , et al. A retrospective analysis to examine factors associated with mortality in Medicare beneficiaries newly diagnosed with multiple myeloma. Curr Med Res Opin. 2016;32(12):1989‐1996. 27532155 10.1080/03007995.2016.1226166

[32] Knudsen LM , Hjorth M , Hippe E ; Nordic Myeloma Study Group . Renal failure in multiple myeloma: reversibility and impact on the prognosis. Eur J Haematol. 2000;65(3):175‐181. 11007053 10.1034/j.1600-0609.2000.90221.x

[33] Gonsalves WI , Godby K , Kumar SK , Costa LJ . Limiting early mortality: Do's and don'ts in the management of patients with newly diagnosed multiple myeloma. Am J Hematol. 2016;91(1):101‐108. 26214377 10.1002/ajh.24129

